# Multistage Regulation Strategy via Fluorine‐Rich Small Molecules for Realizing High‐Performance Perovskite Solar Cells

**DOI:** 10.1002/advs.202412557

**Published:** 2024-12-12

**Authors:** Xiong Chang, Kunpeng Li, Yong Han, Guohua Wang, Zhishan Li, Dongfang Li, Fashe Li, Xing Zhu, Hua Wang, Jiangzhao Chen, Tao Zhu

**Affiliations:** ^1^ Faculty of Metallurgical and Energy Engineering Kunming University of Science and Technology Kunming 650093 P. R. China; ^2^ China Three Gorges Yunnan Energy Investment Co., Ltd Lijiang 650000 P. R. China; ^3^ Faculty of Metallurgical and Energy Engineering/State Key Laboratory of Complex Nonferrous Metal Resources Clean Utilization Kunming University of Science and Technology Kunming 650093 P. R. China; ^4^ Faculty of Materials Science and Engineering Kunming University of Science and Technology Kunming 650093 P. R. China; ^5^ Faculty of Metallurgical and Energy Engineering/Yunnan Key Laboratory of Clean Energy and Energy Storage Kunming University of Science and Technology Kunming 650093 P. R. China

**Keywords:** charge transport, crystallization control, multistage regulation, perovskite solar cells

## Abstract

Perovskite solar cells (PSCs) are an ideal candidate for next‐generation photovoltaic applications but face many challenges for their wider application, including uncontrolled fast crystallization, trap‐assisted nonradiative recombination, and inefficient charge transport. Herein, a multistage regulation (MSR) strategy for addressing these challenges is proposed via the introduction of fluorine‐rich small molecules with multiple active points (i.e., 1‐[Bis(trifluoromethanesulfonyl)methyl]‐ 2,3,4,5,6‐pentafluorobenzene (TFSP)) into the precursor solution of the perovskite film. The addition of TFSP effectively delays and regulates the crystallization and growth process of the perovskite film for larger grains and fewer defects, and it effectively improves the coverage of self‐assembled molecules for efficient charge transport. The multiple active points of TFSP induce a strong binding affinity with uncoordinated defects in the perovskite film. Moreover, the high fluorine content of TFSP induces strong electronegativity to establish a high binding strength between the perovskite film and electron transport layer. Finally, PSCs prepared by the MSR strategy demonstrated an optimal power conversion efficiency (PCE) of 25.46% and maintained 91.16% of the initial PCE under nonpackaged air conditions and at a relative humidity of 45% after 3000 h.

## Graphical abstract

TFSP can form new perovskite intermediates in the precursor solution to delay the solution aging and regulate the crystallization process, inhibit excessive self‐agglomeration of MeO‐4PACz, multi‐site anchor perovskite lattice structure, and enhance the interaction with PCBM. As a result, TFSP achieves an innovative multi‐stage regulation strategy through the precise regulation of different stages of the perovskite device construction.

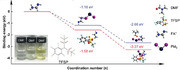

## 1 Introduction

Inverted perovskite solar cells (PSCs) with a p–i–n structure have received much attention in the solar power industry because of their high efficiency, low cost, and easy fabrication process.^[^ ^1^, ^2^, ^3^, ^4^, ^5^, ^6^ ^]^ At present, PSCs have achieved a certified power conversion efficiency (PCE) of >26.7%,^[^ ^7^ ^]^ which is comparable to that of single‐crystal silicon solar cells and demonstrates their potential for mass‐scale manufacturing and commercialization.^[^ ^8^, ^9^, ^10^ ^]^ Despite the high PCE, however, PSCs still exist undesirable features such as uncontrolled fast crystallization, inadequate intrinsic conductivity, mismatched energy levels, poor interfacial contact, and severe carrier charge nonradiative recombination at the perovskite/transport layer interface, which hindered the further improvements of the photovoltaic performance and long‐term stability of PSCs.^[^ ^11^, ^12^, ^13^, ^14^, ^15^, ^16^, ^17^, ^18^, ^19^ ^]^

Various approaches have been applied to address the above issues, such as additive engineering, solvent engineering, and interface engineering.^[^ ^20^, ^21^, ^22^, ^23^ ^]^ Qu et al. used an additive engineering approach of introducing chlorine‐containing organic molecules into the perovskite film to form a capping layer that blocks moisture penetration while preserving complexes based on dimethyl sulfoxide (DMSO) to regulate crystallization.^[^ ^24^ ^]^ Zhang et al. used a solvent engineering approach by applying a crown ether with large ring size and strong electron donor characteristics to suppress the formation of high‐order iodoplumbates and harmful byproducts such as HI and I_3_^−^, which stabilized the perovskite precursors for up to 120 days.^[^ ^25^ ^]^ Liu et al. used an interface engineering approach and reported a molecular hybrid at the buried interface of inverted PSCs by co‐assembling multiple carboxylic acid‐functionalized aromatic compounds with self‐assembled molecules (SAMs) to improve the heterojunction interface.^[^ ^26^ ^]^ Zhan et al. connected [60]fullerenes with different lengths of the flexible alkyl chain as an interval to solve the problem of high energy disorder and insufficient passivation of [6,6]‐phenyl‐C61‐methyl‐butyrate (PCBM), and achieved a PCE of 23.08%.^[^ ^27^ ^]^ However, the development of a single strategy that can simultaneously modify perovskite crystallization, the hole transport layer (HTL), perovskite defects, and the electron transport layer (ETL) is a major challenge.

In this study, we developed a multistage regulation (MSR) strategy by designing a novel material that can be introduced into the perovskite precursor fluid to achieve a pinhole‐free high‐quality perovskite film with well‐covered SAMs and a high charge transport efficiency. The MSR strategy uses 1‐[Bis(trifluoromethanesulfonyl) methyl]‐2,3,4,5,6‐pentafluorobenzene (TFSP), which is a fluorine‐rich small molecule containing multiple active points and possessing outstanding electronegativity and excellent electrical conductivity.^[^ ^28^ ^]^ TFSP optimizes the structure of the PSCs in multiple stages: 1) In the precursor stage, TFSP increases the formation path of perovskite intermediates to generate larger perovskite grains; 2) In the annealing stage, TFSP alleviates excessive self‐assembly of SAMs during recrystallization to improve the charge transport properties of the HTL; 3) In the stabilization stage, the multiple active points of TFSP stabilize the crystal structure and passivate point defects; 4) In the ETL construction stage, the high electronegativity of TFSP binds the upper surface of the perovskite film with the ETL and reduces the mismatch between energy levels. As a result, PSCs prepared by the MSR strategy achieved a champion PCE of 25.46% and maintained 91.16% of the initial PCE under nonpackaged air conditions and at a relative humidity of 45% after 3000 h. The proposed MSR provides a novel and feasible method to enhance the performance of PSCs for wider applications.

## 2 Results and Discussion

**Figure 1:**
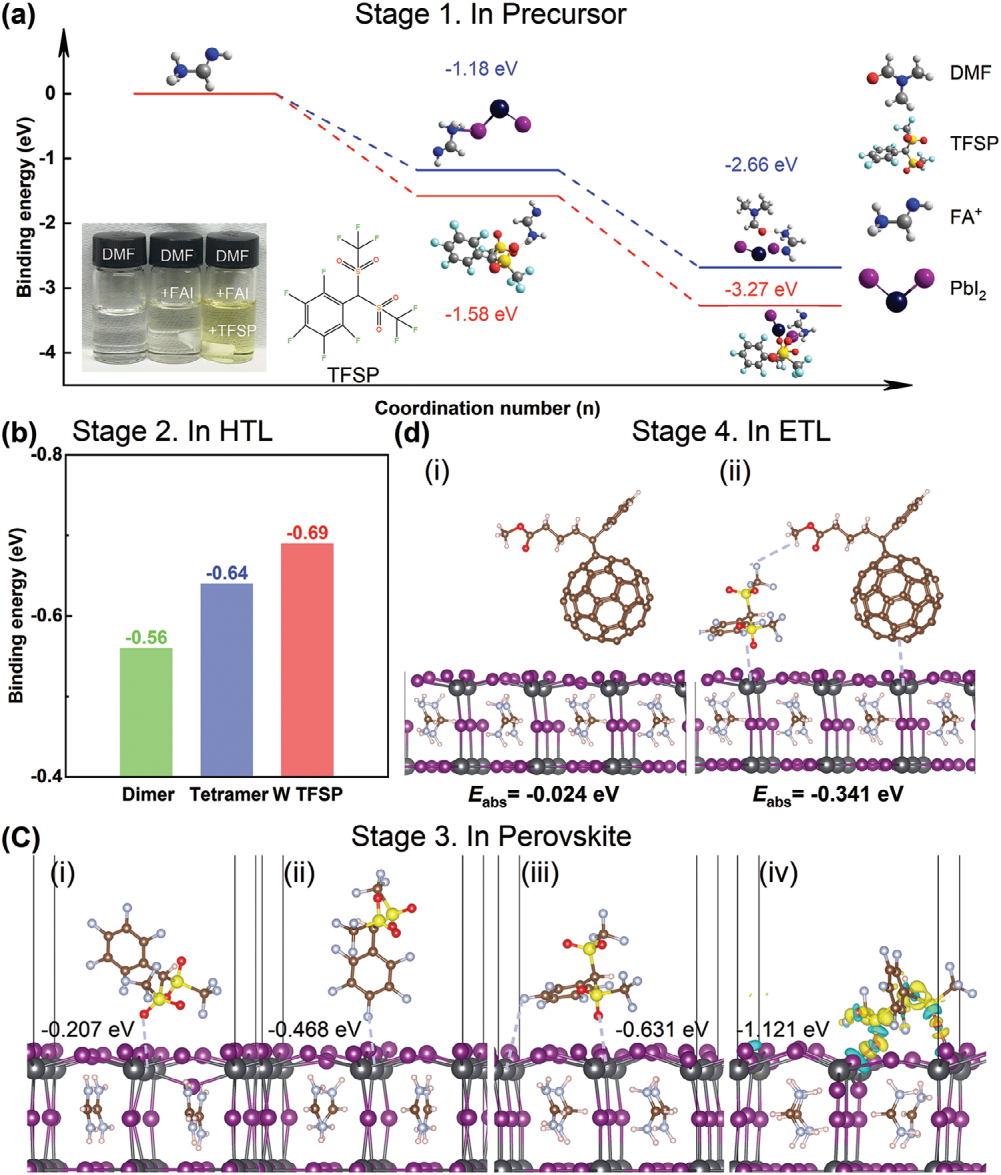
a) Reaction coordinate diagram of formation paths for different perovskite intermediates in DMF. b) Binding energies of MeO–4PACz dimer, MeO–4PACz tetramer, and MeO–4PACz dimer with TFSP. c) Interactions of different configurations of TFSP with the perovskite surface: i) perpendicular to the perovskite surface with the benzene ring above, ii) perpendicular to the perovskite surface with the benzene ring below, and iii) parallel to the perovskite surface; iv) side view of the interactions of TFSP (dashed ovals) with the Pb/I terminated (001) FAPbI_3_ surface. PbI_6_ octahedra are highlighted to show that major structural relaxation was mostly observed in the uppermost layer. Changes in the charge‐density profile around all species involved in the TFSP–surface binding include prominent charge accumulation (yellow) and depletion (blue) regions indicating F─Pb and O─Pb interactions. d) Binding of perovskite layers with and without TFSP to PCBM in the ETL.

The crystallization of a halide perovskite (ABX_3_) for photovoltaic devices usually involves a reaction between a metal halide (BX_2_) and monovalent halide (AX), where X is generally an iodide.^[^ ^29^ ^]^ Because perovskite films are usually treated by solution, an important factor that governs the kinetics of crystallization is the solvent–solute interaction, which determines the structure of the solvation intermediates and thus the activation barrier of the BX_2_–AX reaction. We used density functional theory (DFT) to calculate the reaction enthalpies between PbI_2_, formamidinium iodide (FAI), *N*,*N*‐dimethylformamide (DMF), and TFSP and to determine the formation path of perovskite intermediates (**Figure** **1**a; Figure S1, Supporting Information). For the initial solution system, PbI_2_ has a higher binding strength with FAI than that of either PbI_2_ or FAI with DMF, which is an important factor for the excessively fast crystallization speed of perovskite.^[^ ^30^ ^]^ However, TFSP has a higher binding strength with FAI than PbI_2_ does with FAI, so its introduction not only results in the formation of a new perovskite intermediate but also directly slows the crystallization speed of perovskite. Furthermore, the FAI·TFSP·PbI_2_ intermediate has a better binding strength than the FAI·DMF·PbI_2_ intermediate, which further weakens the aging of the target precursors.^[^ ^31^, ^32^ ^]^ The liquid ultraviolet−visible (UV‐vis) spectroscopy results (Figure S2, Supporting Information) also showed that introducing TFSP increased the intensity of the target solution, which indicates the formation of a larger cluster at the beginning of the perovskite spin–coating stage.^[^ ^33^ ^]^ Therefore, because the BX_2_–AX reaction involves the decomplexation of solvate intermediates, the introduction of TFSP results in a high activation barrier for the crystallization of perovskite, which reduces the activation rate and facilitates the formation of larger grains.

Interestingly, introducing TFSP greatly increases the binding energy of [4‐(3,6‐Dimethoxy‐9*H*‐carbazol‐9‐yl)butyl]phosphonic Acid (MeO–4PACz) self‐assembling dimers because of the coexistence of π−π interaction and hydrogen bonds.^[^ ^15^, ^26^ ^]^ The interaction energy between TFSP and MeO–4PACz (TFSP·MeO–4PACz) dimers (−0.69 eV) is comparable to the hydrogen binding energy (−0.64 eV) of MeO–4PACz tetramers (Figure 1b; Figure S3, Supporting Information). Thus, introducing TFSP facilitates the formation of TFSP·MeO–4PACz dimers, which disrupts the formation of MeO–4PACz tetramers and helps reduce agglomeration to achieve a uniform distribution at the buried interface and thus a tightly uniform NiO_X_/SAM HTL.

Because TFSP has multiple active sites, we investigated how the molecular structure of the ligand affects orientation. The formation energies of three configurations were considered (Figure 1c): i) one ligand perpendicular to the perovskite surface with the benzene ring above, ii) one ligand perpendicular to the perovskite surface with the benzene ring below, and iii) one ligand parallel to the perovskite surface. Although the active sites of TFSP bind to cations on the perovskite surface in both perpendicular configurations, the parallel configuration results in more effective binding with the perovskite surface because the S═O functional group provides an additional bond that can passivate the two active sites of Pb^2+^ (Figure 1c(iv)).

We also investigated the effect of the ligand orientation on the charge transfer at the perovskite interface by using PCBM as the ETL, which is known to cause energy loss in PSCs.^[^ ^34^, ^35^, ^36^ ^]^ The calculated differences in charge density (Figure 1d) indicate that a perovskite layer incorporating TFSP has a notably higher binding strength with the ETL [adsorption energy (*E*_ads_) = −0.341 eV] than a pure perovskite layer does (*E*_ads_ = −0.024 eV). This strengthens the adsorption interaction between perovskite and PCBM, which is beneficial for electron transport.^[^ ^37^ ^]^

Fourier transform infrared spectroscopy (FTIR) was conducted to confirm the above results (Figure S4, Supporting Information). The addition of PbI_2_ shifted the stretching vibration of S═O bonds for the pristine TFSP molecule from 1304.1 to 1306.5 cm^−1^, respectively, which indicates the strong coordinate interactions between O and Pb atoms. Meanwhile, the N─H stretching peaks of FAI shifted from 1630.5 to 1636.7 cm^−1^ after the introduction of TFSP. The characteristic peaks shifted to high wave numbers when fluorine‐containing molecules were mixed with FAI, which was primarily attributed to the strengthened hydrogen bonding by F atoms. Therefore, the TFSP successfully changed FAI and PbI_2_ film morphology (Figure S5, Supporting Information), which demonstrates the interactions of TFSP with FAI and PbI_2_ from the visible scale. X‐ray photoelectron spectroscopy (XPS) was conducted to clarify the interaction mechanism between TFSP and perovskite (Figure S6, Supporting Information). The Pb 4f XPS spectra revealed that the Pb 4f 5/2 and 4f 7/2 peaks of the control perovskite film (i.e., without TFSP) were at 143.12 and 138.28 eV, respectively. For the target perovskite film (i.e., with TFSP), the introduction of TFSP shifted these peaks to lower binding energies, which was ascribed to the interaction between the lone‐pair electrons of the unsaturated groups in TFSP and the undercoordinated Pb^2+^ ions, thus decreasing the electron density around Pb.^[^ ^38^ ^]^ Similarly, the I 3d 3/2 and I 3d 5/2 peaks initially positioned at 630.61 and 619.07 eV, respectively, in the control film shifted to 630.47 and 618.93 eV, respectively, in the target film. These results indicate that TFSP can effectively compensate for halide vacancies within the perovskite matrix.^[^ ^39^ ^]^

**Figure 2:**
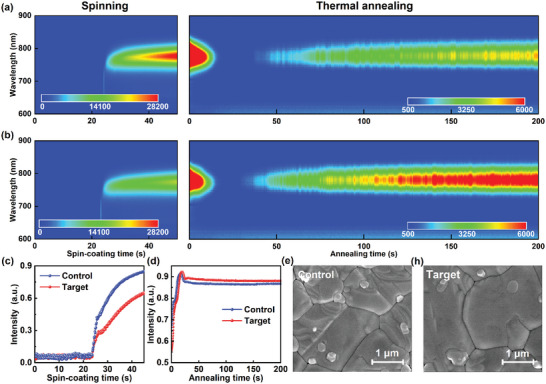
Comparison between control and target films: a, b) in situ PL maps, c, d) in situ UV‐vis intensity of 700 nm, e, f) top‐view Scanning electron microscopy (SEM) images.

In situ photoluminescence (PL) and UV‐vis absorption spectroscopies were conducted to investigate the effects of introducing TFSP on the spin–coating and annealing stages (**Figure** **2**). During the spin–coating stage, a PL peak of perovskite at ≈ 780 nm was immediately observed in both the control and target films after doping of Chlorobenzene (CB) as an antisolvent (Figure 2a,b), and the PL peak corresponding to perovskite continuously redshifted owing to the ongoing increase in the size of the perovskite crystals.^[^ ^40^ ^]^ However, the target film had a substantially lower PL intensity than the control film, which indicates that the former had a slower crystallization process. A spin stage with rapid nucleation and slow crystallization should yield high‐quality perovskite films and thus enhance the photovoltaic performance of PSCs. During the annealing stage, two distinct periods were observed for the film crystallization process. The first was the precursor dissolution period, where the perovskite crystal structure generated by the rotary coating process was destroyed by heating, which reduced the PL intensity. The second was the recrystallization period, where the perovskite recrystallized and grew larger grains, which increased the PL intensity and improved the crystallinity. This period continued until the end of the annealing stage. The target film exhibited greater PL intensity quenching throughout the recrystallization period than the control film, which indicates enhanced electron extraction and transportation at the buried interfaces. In situ, UV‐vis maps (Figure S7, Supporting Information) were conducted, and representational in situ UV‐vis absorption curves of control and target perovskite films at 700 nm (Figure 2c,d) also exhibited similar results with in situ PL maps.

The effect of TFSP on the surface morphology was investigated through SEM measurement (Figure 2e,f). Adding TFSP greatly increased the perovskite grain size and reduced the sizes of the white phases at the grain boundaries, which have been proven to be incompletely reacted PbI_2_.^[^ ^41^ ^]^ The target film had an average grain size of 2.2 µm, which is substantially larger than the grain size of 1.2 µm for the control film (Figure S8, Supporting Information) and indicates a clear enhancement in crystallinity. The cross‐sectional SEM images (Figure S9, Supporting Information) reveal that the target film had a smoother interface between the perovskite layer and HTL, which was attributed to the reaction between TFSP and uncoordinated Pb and/or Pb clusters on the perovskite surface. Atomic force microscopy (AFM) was used to examine the surfaces of the control and target films (Figure S10, Supporting Information). The target film had a root‐mean‐square (RMS) value of 20.1 nm while the control film had an RMS value of 28.7 nm, which means a lower surface roughness. The SEM and AFM results demonstrate that the target film had a smoother and neater surface with fewer defects, which can effectively suppress carrier recombination at the perovskite/PCBM interface and improve the short‐circuit current density (*J*_SC_) and fill factor (FF).^[^ ^42^, ^43^ ^]^

To examine the differences in surface energy of the perovskite grains for the control and target films, the theoretical calculation DFT of binding energies between TFSP and Pb atoms across various crystal facets—namely, (100), (111), and (110) in FAPbI_3_, the primary constituent of perovskite (Figure S11, Supporting Information). DFT results show that TFSP shows a stable binding energy of −0.63 eV on the (100) facet of FAPbI_3_, compared to the (111) and (110) facets (−0.18 and −0.20 eV). This preferential combination of DAT molecules on the (100) facet of FAPbI_3_ indicates the potential decrease of surface energy and the enhancement of thermodynamic driving force, thus inducing a preferential crystal orientation in perovskite along the (100) facet.^[^ ^44^, ^45^ ^]^ The X‐ray diffraction (XRD) patterns (Figure S12a, Supporting Information) demonstrated that the addition of TFSP greatly enhanced the (100) peak intensity, which is consistent with the DFT calculations. The target film had a narrower full width at half‐maximum and higher intensity of the (100)‐oriented peak (14.1°) compared with the control film (Figure S12b, Supporting Information). Thus, adding TFSP increased the peak intensities for the signals representing the (100) and (200) crystal planes by 24% and 13%, respectively. Therefore, introducing TFSP effectively converts undesirable PbI_2_ to favorable perovskite crystals for improved crystallinity, larger grains, and fewer grain interface defects. The bulk defects in the perovskite film are reduced, and carrier recombination is inhibited.

**Figure 3:**
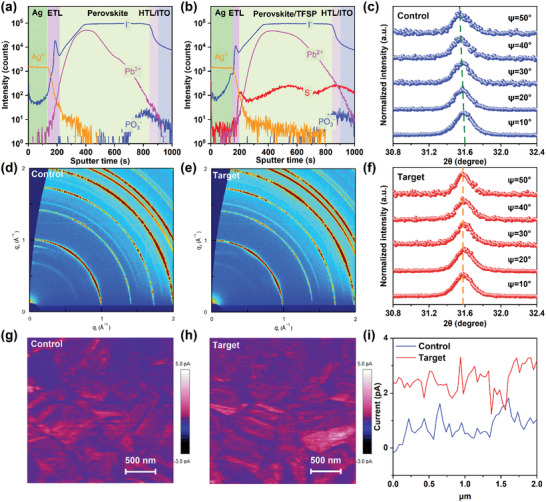
ToF‐SIMS of a) control and b) target films. c, f) GIXRD with different ψ angles (10–50°) at a depth of ≈200 nm. 2D GIWAXS maps of d) control and e) target films. cAFM maps of g) control and h) target films. (i) Current as a function of distance for the control and target films obtained from the cAFM maps.

Time‐of‐flight secondary ion mass spectrometry (ToF‐SIMS) was used to investigate the distribution of TFSP molecules in the target film (**Figure** **3**a,b). The TFSP molecules (represented by the unique S^−^) showed a homogenous distribution in the bulk perovskite and at the MeO‐4PACz/perovskite/PCBM interfaces. The target film had a more uniform Pb distribution than the control film. Therefore, the TFSP molecules demonstrated a holistic defect passivation effect on the top surface and perovskite bulk through molecular interaction between their functional groups. Furthermore, the TFSP showing diffusion into the HTL and ETL in ToF‐SIMS results corroborates the existence of interactions with the HTL and ETL. Thus, the static contact angles were measured for the precursors of the control and target films on ITO/MeO‐4PACz substrates and were determined to be 16 and 9°, respectively (Figure S13, Supporting Information). These results indicate that TFSP improves the wettability of the perovskite film for better coverage of the substrate.^[^ ^46^ ^]^ Moreover, the effects of TFSP on the conductivity and carrier mobility were investigated (Figures S14, S15, Supporting Information). The introduction of TFSP increased the conductance of MeO‐4PACz and PCBM from 9.3 × 10^−3^ and 6.4 × 10^−3^ to 1.24 × 10^−2^, and 7.2×10^−3^ mS cm^−1^, respectively. The carrier mobilities of the MeO‐4PACz and PCBM layers were obtained by the Hall effect test and increased from 100 and 112 to 106 and 145 cm^2^ V^−1^·s^−1^, respectively, after the treatment of the TFSP. The higher carrier mobility for the MeO‐4PACz/TFSP and PCBM/TFSP layer indicates that it can extract electrons generated by perovskite more quickly. Fluorescence intensity was further analyzed by PL for the ITO/MeO‐4PACz/ Perovskite and ITO/Perovskite/PCBM stack structures. As expected, the treated films both showed lower PL intensity and suggesting that FPA enhances carrier extraction (Figure S17, Supporting Information), which demonstrates the effectiveness of the multistage regulation strategy for HTL/ETL.

The significant improvement in the morphology and crystallinity of the perovskite at the bottom region also affects the release of residual stress.^[^ ^47^ ^]^ We thus investigated the residual stress at the bottom surface of the different perovskite films via the grazing incidence X‐ray diffraction (GIXRD) technique with the 2θ−sin^2^ ψ method with a penetration depth of 100 nm (Figure 3b,f). It was found that the diffraction peaks of the control perovskite film gradually shifted to lower 2θ positions by varying ψ from 10 to 50°, while the diffraction peaks of the target perovskite film slightly shifted to higher 2θ positions. The fitting lines of 2θ−sin^2^ ψ (Figure S17, Supporting Information) reveal that the target film had a lower slope value (−0.027) than the control film (−0.144). The control perovskite films exhibited negative slopes, indicating that the films were subjected to tensile stress. In contrast, the slope of the target perovskite film showed a diminutive positive value, suggesting a favorable situation with slight compressive stress. These results indicate that the MSR strategy can release the residual tensile stress of perovskite films, which is favorable for the efficiency and stability of PSCs.^[^ ^48^ ^]^

Grazing incidence wide‐angle X‐ray (GIWAXS) data of the control and target films (Figure 3d,e) indicated that the most prominent scattering ring was at the momentum transfer (q) ≈ 1 Å^−1^, which can be indexed to the (100) crystal plane. Meanwhile, weak scattering signals were detected at q = 0.9 Å^−1^, which indicates the slight presence of PbI_2_ residuals. These observations prove that the main constituents of the perovskite crystal structure remained unaltered upon the addition of TFSP. However, a slight decrease in q for the characteristic (100) peak after TFSP addition (Figure S18, Supporting Information) is indicative of lattice expansion.^[^ ^9^, ^49^ ^]^ This may be due to the effective passivation of V_I_ defects by TFSP, which relaxes the local lattice strain and effectively improves the interface contact between the perovskite and HTL.^[^ ^50^, ^51^ ^]^

PL mapping was used to analyze the optical properties of the film surfaces (Figure S19, Supporting Information). The target film exhibited a heightened fluorescence intensity and more uniform emission than the control film. In addition, conductive AFM (cAFM) was used to obtain the current as a function of distance (Figure 3g,h). Compared with the control film, the target film exhibited a smoother morphology and higher grain‐interior and lower grain‐boundary currents (Figure 3i), which would facilitate vertical carrier transport.^[^ ^52^ ^]^

**Figure 4:**
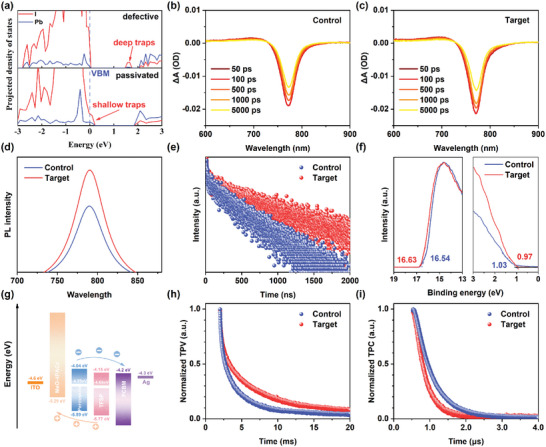
a) Projected density of states of the V_I_ defective perovskite slab passivated without and with TFSP. Transient absorption (TA) spectra at different delay times for the b) control and c) target films. d) Steady‐state PL spectra and e) TRPL decay curves of the control and target films. f) Ultraviolet photoelectron spectroscopy (UPS) spectra of secondary‐electron cutoff (left) and valence band (right) regions for the control and target films. g) Energy‐level alignment of ITO/MeO‐4PACz/perovskite with and without TFSP/PCBM/Ag. g) TPV and h) TPC decay curves of control and target films.

To assess the ability of TFSP to passivate the deep‐level defects near the Fermi level in the perovskite, the respective projected density of states (PDOS) of the 𝛼‐FAPbI_3_ perovskite structure with and without TFSP were compared (**Figure** **4**a). For all surface‐defect‐containing pristine systems (V_I_, Pb_I_, and V_Pb_), the appearance of trap states near the Fermi level of the perovskite surface can be easily identified.^[^ ^53^ ^]^ Notably, these trap states disappear after TFSP treatment, indicating that the bonds formed between TFSP and perovskite are beneficial to eliminating the trap states of the perovskite surface. Since these deep‐level defects act as the defect centers that result in nonradiative recombination, the effective passivation of these deep‐level defects by TFSP is beneficial to reduce the nonradiative recombination intensity of perovskite film. Therefore, the TA spectra revealed that the target film had a lower quenching rate of the ground state bleaching signal than the control film (Figure 4b,c; Figure S20, Supporting Information), which provides further support that introducing TFSP into perovskite films suppresses nonradiative recombination. Moreover, the PL intensity of the target film excited from the perovskite side was 24% higher than that of the control film (Figure 4d), which indicates that TFSP effectively reduces the trap density and thus minimizes nonradiative recombination in perovskite films. Time‐resolved photoluminescence (TRPL) was used to study the carrier transport and recombination in the perovskite layer, which related to the domination of second‐order trap‐assisted recombination (Figure 4e). Adding TFSP increased the carrier charge lifetime of the perovskite film dramatically from 774 to 1427 ns, facilitating interface carrier charge extraction.

To investigate the effect of TFSP on the surface energy and energy level of perovskite films, a series of characterizations was carried out. Kelvin probe force microscopy results (Figure S21, Supporting Information) showed that the target film had a more uniform potential distribution with a range of −219 ± 51 mV while the control film had a potential distribution of −68 ± 60 mV. The decreased surface potential was attributed to the high electronegativity of TFSP, and the narrower distribution was attributed to a more homogeneous surface. Thus, TFSP was shown to reduce the number of defects. UV‐vis spectroscopy of the perovskite films demonstrated that both the target and control films had absorption edges at ≈808 nm (Figure S22a, Supporting Information), which means excellent absorption ability. A Tauc plot was generated (Figure S22b, Supporting Information), and both the control and target films had a bandgap (*E*g) of 1.55 eV, which is suitable for light absorption. The target film showed an enhanced absorption intensity that was attributed to the improved crystallinity. The UPS results indicated that TFSP changed the surface energy of the perovskite film, and they were used to map the secondary‐electron cutoff and valence band regions (Figure 4f). The valence band (*E*_VB_) was determined by *W*_F_ = 21.22 eV − *E*_cutoff_ and *E*_VB_ = *W*_F_ + VBM, where *W*_F_ and VBM are the work function and valance band maximum, respectively. Adding TFSP was found to increase *W*_F_ from 4.59 to 4.68 eV, and the vacuum level was negligible (*E*_VAC_ = 0 eV). Meanwhile, adding TFSP decreased VBM from −5.59 to −5.77 eV (Table S1, Supporting Information). Because the bandgap determined from the UV‐vis spectra remained almost the same between the control and target films with a value of 1.55 eV, these results indicate that the conduction band minimum followed the same downward shift. The energy‐level alignment diagram (Figure 4g) indicates a downward shift of energy bands from the control film to the target film, which is beneficial for the carrier extraction and charge transfer from the perovskite layer to the ETL.^[^ ^54^ ^]^

To determine the origin of the improved charge transport dynamics with the MSR strategy, the optical and electrical characteristics of the perovskite films were comprehensively investigated by transient photovoltage spectrum (TPV) and transient photocurrent spectrum (TPC) (Figure 4h, i). The TPV curves indicated that the target film had a longer lifetime (5.94 ms) than the control film (4.23 ms) and exhibited the characteristics of a stretched exponential decay, which is typical of complex first‐order trapping and recombination processes involving electron transport among defect states at grain boundaries or interlayer interfaces. The TPC curves indicated that the target film had a much shorter lifetime (1.37 µs) than the control film (1.98 µs). The fast decay of the photocurrent indicates that the target film had a better charge carrier mobility, fewer traps, and higher doping intensity.

**Figure 5:**
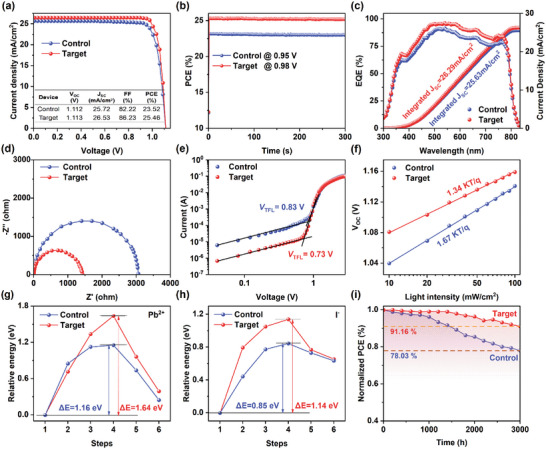
a) Photocurrent density−voltage (*J*−*V*) curves and b) steady‐state output of control and target PSCs with an active area of 0.043 cm^2^. c) External quantum efficiencies (EQE) spectra of control and target PSCs. d) Nyquist plots of the Electrical impedance spectra (EIS) for control and target PSCs at *V* = 0.9 V. e) Dark *I–V* curves for hole‐only (ITO/MeO‐4PACz/perovskite/Ag) devices based on the control and target PSCs. f) Influence of the light intensity on *V*_OC_ of PSCs. Energy profiles of Pb^2+^ and I^−^ ion migration in g) control and h) target PSCs. The structures of the nudged elastic band (NEB) images in the initial, transition and final states are shown. i) Normalized PCE decay of control and target PSCs stored in ambient air.

PSCs featuring a p–i–n structure were fabricated to evaluate the effects of TFSP on the device performance. The structure comprised nickel oxide and MeO‐4PACz as the HTL, PCBM as the ETL, and Bathocuproin (BCP) as the hole block layer (Figure S23, Supporting Information). Compared with the control PSC (i.e., without TFSP), the target PSC (i.e., with TFSP) showed an increase in PCE from 23.32% to 25.46% (**Figure** **5**a). The PCE and short‐circuit current density (*J*_SC_) histograms of the control and target PSCs were obtained along with the statistical distributions of J_SC_, the open‐circuit voltage (*V*_OC_), and FF that determine the PCE (Figure S24, and Table S2 Supporting Information). As expected from the defect concentration and carrier lifetime analyses of the perovskite films, the target PSC had a much higher average PCE than the control PSC. The improved PCE for the target PSC was attributed to increases in *J*_SC_ and FF. The average J_SC_ improved from 25.28 mA cm^−2^ for the control PSC to 26.31 mA cm^−2^ for the target PSC while FF improved from 82.07% to 85.34%. The target PSC demonstrated a high PCE of 25.22% for the reverse scan (*J*_SC_ = 26.37 mA cm^−2^, *V*_OC_ = 1.114 V, and FF = 85.85%) and 24.86% for the forward scan (*J*_SC_ = 26.10 mA cm^−2^, *V*_OC_ = 1.113 V, and FF = 85.56%) for a negligible hysteresis of 1.0%. In contrast, the control PSC demonstrated a hysteresis of 2.9% (Figure S25 and Table S3, Supporting Information).

The target PSC achieved a steady‐state PCE of 25.25% for 300 s under continuous maximum power point while the control PSC achieved a steady‐state PCE of 24.17% (Figure 5b). The typical EQEs of the control and target PSCs were measured, and the integrated currents matched well with the *J*_SC_ values obtained from the *J*–*V* curves (Figure 5c). The *J–V* curves under dark conditions (Figure S26, Supporting Information) indicated that the target device had a reduced leakage current, which corresponds to a reduced charge recombination and higher carrier generation rate and is associated with the trap density. EIS was obtained to clarify the carrier transport and recombination properties (Figure 5d). The target PSC exhibited a much lower charge transfer resistance (*R*_ct_) than the control PSC, which suggests that TFSP enhances the charge transport and reduces the charge recombination of perovskite films. To clarify the factors contributing to the enhanced *V*_OC_, the Mott–Schottky (M–S) plots and built‐in potential (*V*_bi_) of the PSCs were obtained (Figure S27, Supporting Information). The increased *V*_bi_ for the target PSC indicates that the built‐in band alignment contributed a stronger driving force for the intrinsic charge separation. In addition, the carrier density at the interface is inversely proportional to the slope value of the linear regime. The target PSC had a lower carrier density, which indicates a faster and more effective charge transfer at the perovskite/HTL interface.

Space charge limited current measurements were performed to investigate the trap density (*N*_trap_) of perovskite films. Hole‐only devices with a configuration of ITO/MeO‐4PACz/perovskite/MeO‐4PAC/Ag and electron‐only devices with a configuration of ITO/PCBM/perovskite/PCBM/Ag (Figure 5e; Figure S28, Table S4, Supporting Information) were fabricated. Adding TFSP was shown to reduce the electron defect density from 4.48 × 10^15^ to 3.34 × 10^15^ cm^−3^ and the hole defect density from 1.37 × 10^16^ to 1.12 × 10^16^ cm^−3^. The decreased quantity of electron and hole traps confirmed that TFSP efficiently mitigated the carrier defect states of the perovskite film.

The double logarithmic relationship between *J*_SC_ and the light intensity was calculated based on the proportional relationship between the light intensity power exponent and *J*_SC_. An ideal device free from bimolecular recombination involving free carriers should exhibit an α value close to unity. Adding TFSP decreases deep trap states by passivating grain boundaries, which allows for more effective extraction of charge carriers. The target PSC achieved a larger α value of 0.993 compared to the control device, which had an α value of 0.986 (Figure S29, Supporting Information). The relationship between the light intensity and V_OC_ was utilized to evaluate the diode characteristics of the PSCs. Because *J*_SC_ is directly proportional to the light intensity and greatly exceeds *J*_0_, *V*_OC_ is proportional to the logarithm of the light intensity with a slope of *nkTq* ^−1^. Typically, *n* is equal to one without trap‐assisted carrier recombination. The control PSC exhibited *n* = 1.67 whereas the target PSC had a lower *n* = 1.33 (Figure 5f), which indicates that TFSP suppresses trap‐assisted nonradiative recombination at the interface to increase *V*_OC_. These findings are consistent with the TRPL analysis of the perovskite films.

Finally, we investigated the long‐term operational stability of the control and target PSCs. The aging UV‐vis spectra under light conditions (Figure S30 Supporting Information) indicated that the control PSC had a rapid decrease in absorption intensity and greatly deformed absorption curve while the target PSC maintained a basically constant absorption intensity and undeformed absorption curve after 4 weeks in ambient environment. Thus, the target PSC demonstrated superior stability under light conditions. To clarify how TFSP improves the device stability, DFT calculations were performed using the NEB method to model the Pb^2+^ and I^−^ migration pathway and calculate the activation energy barrier (∆*E*_a_) for ion migration (Figure 5h,i). The *∆E*_a_ values of Pb^2+^ and I^−^ demonstrated prominent enhancement of 0.48 and 0.39 eV, respectively, attributing to the introduction of TFSP, which indicated that TFSP inhibits decomposition. The higher ∆*E*_a_ may be attributed to the electrostatic interactions and steric hindrance induced by TFSP on the perovskite surface, which suppresses the migration of Pb^2+^ and I^−^ ions.^[^ ^55^ ^]^ The contact angle between the perovskite film and H_2_O was 70° for the control PSC but increased to over 77° for the target PSC (Figure S31, Supporting Information), which indicates that the improved water resistance can be attributed to TFSP, which is rich in hydrophobic groups. The stability of the PSCs was investigated at a relative humidity of 45%. Despite being uncovered, the target PSC retained 91.16% of its initial PCE after operating in an ambient environment for 3 000 h (Figure 5g). In contrast, the control PSC decreased to 78.03% of its initial PCE. The enhanced operational stability can be attributed to TFSP decreasing the trap density and impurities (including excess PbI_2_ and nonperovskite polytypes) on the surface and in the perovskite bulk.

## 3 Conclusion

The proposed MSR strategy introduces TFSP to the perovskite precursor solution to improve the PSC performance by improving the crystallinity, inhibiting defects, enhancing interfacial contact, and reducing mismatch in energy levels. The fabricated PSC demonstrated improved quality and stability, lower interlayer carrier loss and nonradiative recombination strength, and faster interfacial carrier extraction speed. The PSC with TFSP achieved a high PCE of 25.46% and retained 91.16% of the initial PCE under ambient conditions and at a relative humidity of 45% after 3 000 h of operation. The proposed MSR offers a novel and easy approach to improving the performance of perovskite photovoltaic devices for wider application.^[^ ^56^, ^57^, ^58^, ^59^ ^]^

## Conflict of Interest

The authors declare no conflict of interest.

## Supporting information



Supporting Information

## Data Availability

The data that support the findings of this study are available in the supplementary material of this article.
