# Supplementary material for: Multistage Regulation Strategy via Fluorine‐Rich Small Molecules for Realizing High‐Performance Perovskite Solar Cells
Source: Adv Sci (Weinh). 2024 Dec 12;12(5):2412557. doi: 10.1002/advs.202412557 (PMC11791946; doi:10.1002/advs.202412557)
Supplement: Supplementary file 1 — Supporting Information [file ADVS-12-2412557-s001.docx]

**Supporting Information**

**Multistage Regulation Strategy via Fluorine-Rich Small Molecules for Realizing High-Performance Perovskite Solar Cells**

*Xiong Chang, Kunpeng Li, Yong Han, Guohua Wang, Zhishan Li, Dongfang Li, Fashe Li, Xing Zhu*, Hua Wang*, Jiangzhao Chen*, Tao Zhu**

X. Chang, K. Li, D. Li, F. Li, X. Zhu

Faculty of Metallurgical and Energy Engineering

Kunming University of Science and Technology

Kunming, 650093, P. R. China

E-mails: zhuxing2010@hotmail.com (XZ)

Y. Han, G. Wang

China Three Gorges Yunnan Energy Investment Co., Ltd.

Lijiang, 650000, P. R. China

H. Wang

Faculty of Metallurgical and Energy Engineering/State Key Laboratory of Complex Nonferrous Metal Resources Clean Utilization

Kunming University of Science and Technology

Kunming, 650093, P. R. China

J. Chen

Faculty of Materials Science and Engineering

Kunming University of Science and Technology

Kunming, 650093, P. R. China

T. Zhu

Faculty of Metallurgical and Energy Engineering/Yunnan Key Laboratory of Clean Energy and Energy Storage

Kunming University of Science and Technology

Kunming 650093, P. R. China

E-mails: zhutao3306@163.com (TZ)

Keywords: Perovskite solar cells, Charge transport, Crystallization control, Multistage regulation

**Experimental Section**

**Materials**

ITO (high light transmittance, 7 Ω), PCBM (99.9%), nickel oxide (NiO_X_, 99.5%) purchased from Advanced Election Technology. Lead iodide (PbI_2_, 99.9%), Methylammonium iodide (MAI, 99.5%), Methyl Ammonium bromide (MABr, 99.5%), [4-(3,6-dimethoxylate-9H-carbazole-9-base) ethyl] phosphonic acid (MeO-4PACz, 99.5%), cesium iodide (CsI, 99.5%), formamidinium iodide (FAI, 99.5%), lead bromide (PbBr_2_, 99.5%), methylammonium chloride (MACl, 99.5%), purchased from Xi’an Polymer L.T. *N*,*N*-Dimethylformamide (DMF, anhydrous, 99.9%), Dimethyl sulfoxide (DMSO, anhydrous, 99.9%), Chlorobenzene (CB, anhydrous, 99.8%), ethanol (anhydrous, 99.8%), isopropanol (anhydrous, 99.99%), phenethylamine hydroiodide (PEAI, 99.99%) bathocuproin (BCP, 99.99%), 1-[Bis(trifluoromethanesulfonyl) methyl]-2,3,4,5,6-pentafluorobenzene (TFSP, 99.5%) were purchased from Sigma Aldrich. Silver particles were purchased from Beijing VNANO Vacuum Technology Co., Ltd. All materials were used as received without further treatment.

**Device fabrication**

Preparation of Cs_0.05_(FA_0.95_MA_0.05_)_0.95_Pb(I_0.95_Br_0.05_)_3_ perovskite precursor solution: CsI (18.20mg), PbI_2_ (628.50mg), FAI (217.28mg), PbBr_2_ (26.33mg), MABr (7.45mg), MACl (9.45mg) were dissolved in a mixed solvent of 800 μL DMF and 200 μL DMSO (volume ratio 4:1) and stirred for half an hour until all deposits received a yellow transparent 1.4M control perovskite precursor solution. The solution was filtered and rested for light for 1 hour.

Preparation of target perovskite solution: The 1 mmol TFSP powder was dissolved in 1ml DMF solution, heated and stirred at 60℃ for 1 hours to prepare a stock solution of 1 mmol/ml. The 10 μL TFSP reserve solution was mixed with 790 μL of DMF and 200 μL of DMSO to form a TFSP-doped solution at a concentration of 0.1 mmol /ml. Then CsI (18.20mg), PbI_2_ (628.50mg), FAI (217.28mg), PbBr_2_ (26.33mg), MABr (7.45mg), MACl (9.45mg) were dissolved in the 1 ml TFSP-doped solution and stirred for half an hour until all deposits received a yellow transparent 1.4M target perovskite precursor solution. The solution was filtered and rested for light for 1 hour.

Preparation of perovskite solar cells

ITO/NiOx/MeO-4PACz/Cs_0.05_(FA_0.95_MA_0.05_)_0.95_Pb(I_0.95_Br_0.05_)_3_/PEAI/PCBM/BCP/Ag has a typical planar structure, prepared as follows: The ITO glass was sonicated in detergent, deionized water, isopropanol, and ethanol for 15 min. After drying, the ITO glass was treated with ultraviolet ozone (UVO) for 30 min. 65 μL of NiOx solution (10mg/mL in deionized water) was spin-coating onto ITO glass at 2000 rpm and 1000 rpm/s spin acceleration spin coating for 30s, followed by annealing at 120 ℃ for 10 min. Then 40 μL of MeO-4PACz solution (0.5 mg/mL in ethanol) was deposited onto ITO glass at 6000 rpm and 3000 rpm/s spin acceleration spin coating for 35s, followed by annealing at 100℃ for 10 min. After cooling to the MeO-4PACz coated substrate to room temperature (RT), Cs_0.05_(FA_0.95_MA_0.05_)_0.95_Pb(I_0.95_Br_0.05_)_3_ film or TFSP-doped film was constructed from a two-step reverse solvent spin coating using 50 μL precursor liquid evenly over the MeO-4PACz coating, rotating at 2000 rpm and 1000rpm / s and then 4000 rpm and 2000 rpm/s for 30s, and 150 μL chlorobenzene 10s before the end. After spin-coating, the film was heat-annealed at 100 ℃ for 60 min and then cooled to room temperature (RT) for further use. Subsequently, after cooling the substrate to room temperature (RT), the 50 μL PEAI was cast for 30 seconds at a rotational acceleration of 1500rpm and 1000 rpm/s and annealed at 100 ℃ for 5 min. the PCBM was cast for 50 seconds at a rotational acceleration of 1500rpm and 1000 rpm/s without the need for annealing. Subsequently, BCP drops 1 drop (about 7 μl) from 1 mg/ml ethanol solution at a rotational speed of 6000rpm and 3000 rpm/s without annealing. IPSCs was accomplished by the Ag method with a thermal deposition thickness of ~120 nm at the top of the BCP layer under vacuum. The measured effective area of the device was 0.04 cm^-2^.

**Characterization**

X-ray diffraction (XRD) was measured with PANalytical Empyrean X-ray diffractometer with Cu Kα radiation at the scan rate of 5° min^−1^. Steady-state photoluminescence (PL) spectra was carried out with FluoroMax+ R928P. Time-resolved photoluminescence spectroscopy (TRPL) of the film were excited by a 485 nm pulse laser with a repetition frequency of 500 kHz provided by a picosecond pulsed diode laser (FLS980, Edinburgh Instruments Ltd), and the pulse width was 118.6 ps. X-ray photoelectron spectroscopy (XPS) data and ultraviolet photoelectron spectroscopy (UPS) data were obtained through Kratos AXIS ULTRA DLD spectrometer. Non monochromatic He I (21.22 eV) source was used as an excitation source. Gold was used as a reference. The bias voltage applied during the test was ‒9 V. Ultraviolet-visible (UV-vis) absorption spectra were taken on a SHIMADZU UV-2600 spectrophotometer. The surface roughness of perovskite film was measured by an AFM (Nanoscope V, Vecco) in tapping mode. Femtosecond transient absorption spectrum was characterized by an ultrafast spectroscopic system. A Ti: sapphire amplifier (Coherent Co.) supplied laser beams centered at 800 nm with pulse duration of 25 fs, pulse repetition rate of 1 KHz, and a maximum pulse energy of 4 mJ. The output of the amplifier was split into two streams of pulses with a beam splitter. Residual stream was directed into Helios pump-probe system (Ultrafast Systems) to generate the white light continuum probe beam. HRTEM measurements were performed in a JEM-F200. SEM surface and cross-sectional morphology of the films and devices were investigated by scanning electron microscopy (SEM, Hitachi S-4800 microscope). For the cross-sectional image, cross-sectional surface of the sample was coated with ca. 1 nm-thick gold by using sputter to enhance the conductivity. Contact angle tests were implemented on the Drop Shape Analyzer (DSA100, KRÜSS) in static mode at room temperature. AFM and KPFM measurements were obtained using a dimension icon scanning probe microscope (Bruker Dimension Icon) in the tapping mode. ToF-SIMS measurements were performed with a time-of-flight secondary ion mass spectrometer (ToF-SIMS 5, ION-TOF GmbH). Bi ion was used as an analysis source. In situ PL spectra was recorded by a QE-Pro (Ocean Optics) spectrometer with an RPB-785 optical probe located ca. 8 mm above the substrate. Transient absorption (TA) spectra were acquired using a HARPIA spectrometer system. GIXRD patterns were acquired in the air by using a Rigaku Smartlab with Cu Kα radiation in the 2θ range of 30.6°~32.6°. The GIWAXS analysis was conducted at TPS 25A1 within NSRRC. It utilized an incident X-ray photon energy of 15 keV with a beam size of 5 μm by 5 μm. The sample-to-detector distance was approximately 100 mm. The diffraction patterns were captured in two dimensions using a single-photon counting detector, the Eiger X 1M, with a pixel size of 75 μm. The impedance spectrum was measured by using an electrochemical workstation at a bias of 1 V. *J*–*V* curves of the PSCs were measured in the N_2_ glove box by using a Keithley 2400 source meter. An AM 1.5G solar simulator (IQE-200, Newport) and a white light LED (S2000, HECHO) calibrated by a standard silicon solar cell were used as a light source. The devices were measured both in reverse scan (1.2–0 V) and forward scan (0–1.2 V) with a scanning rate of 0.2 V s^−1^. Other scanning rates were also adopted for comparison. The EQE curve was recorded by an Enli Technology (Taiwan) EQE measurement system. A calibrated silicon diode with the known spectral response was used as a reference. The EIS and Mott-Schottky characteristics was measured using CHI600E. The transient photovoltage (TPV) and transient photo- current (TPC) were measured at zero bias in the dark using a nanosecond laser of 532 nm excitation (EKSPLA, NT342A-10) as a small perturbation.

**Computational details**

**Optimization of TFSP structure**

We elected representative repeat unit models from the TFSP and performed calculations on them. Density functional theory (DFT) calculations were conducted by using the Gaussian 16 program at the B3LYP/6-311G* level to obtain the optimized geometries. Based on these optimized structures, we determined the frontier molecular orbital (FMO) and electrostatic potential (ESP) of the respective systems.

**Establishment of TFSP-perovskite supercells**

All the calculations were performed in the framework of the density functional theory (DFT) with the projector augmented plane-wave method, as implemented in the Vienna ab initio simulation package. The generalized gradient approximation proposed by Perdew-Burke-Ernzerhof (PBE) was selected for the exchange-correlation potential. The model was constructed based on the (100) crystal plane of FAPbI_3_ with a 4*4*1 supercell. The cut-off energy for plane wave was set to 500 eV. The energy criterion was set to 10^−5^ eV in iterative solution of the Kohn-Sham equation. All the structures were relaxed until the residual forces on the atoms have declined to less than 0.02 eV/Å. To avoid interlaminar interactions, a vacuum spacing of 20 Å was applied perpendicular to the slab.

The surface energy can be defined by the equation:

|  |  | (1) |
| --- | --- | --- |

In the equation 1, $E_{\text{slab}}^{\text{hkl,σ}}$ was the total energy of slab materials, $E_{\text{bulk}}^{\text{hkl,σ}}$ was the total energy of bulk materials, stands for $E_{\text{slab}}^{\text{hkl,σ}}$ multiple of $E_{\text{bulk}}^{\text{hkl,σ}}$, was the total area of slab materials.

The combination energies (*E*_com_) are calculated as follow:

|  | $\Delta E_{\text{com}}\text{=}E_{\text{A+B}}-E_{\text{A}}-E_{\text{B}}$ | (2) |
| --- | --- | --- |

where $E_{A+B}$ was the total energy of slab A model with B adsorption, $E_{A}$ was the energy of a A slab, and $E_{B}$ was that for a B molecule.

Here, we defined as the charge density difference of A/B heterostructure, where , and were the charge densities of A/B heterostructure, isolated A and B slabs, respectively.


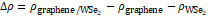

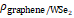

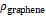

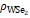


**Analysis of space-charge-limited current (SCLC) model**

The dark *J-V* curves of the hole-only devices can be divided into three parts: Ohmic region, trap-filling limited region with a sharp increase in current and the trap-free Child's region. From eq.3

|  | $J=\frac{9}{8}\mu\varepsilon_{0}\varepsilon\frac{V^{2}}{D^{3}}$ | (3) |
| --- | --- | --- |

The carrier mobility ($\mu$) can be derived to

|  | $\mu=\frac{8d^{3}}{9\varepsilon_{0}\varepsilon}\times\frac{J}{V^{2}}$ | (4) |
| --- | --- | --- |

Where $\frac{J}{V^{2}}$ is the slope of Child’s region. The trap density (*Nt*) of perovskite films can be calculated by eq.4

|  | $N_{t}=\frac{2\varepsilon\varepsilon_{0}V_{TFL}}{qd^{2}}$ | (5) |
| --- | --- | --- |

Where *q* is the electron charge, *d* is the thickness of perovskite film, $\varepsilon_{0}$and$\varepsilon$are the vacuum permittivity and relative dielectric constant of perovskite, and *V_TFL_* is ordinate of the intersection of ohmic region and trap-filling limited region.

VOC can be derived by setting the current to zero in the Shockley equation:

|  | V_OC_ =nkTlnL∕q | (6) |
| --- | --- | --- |

Where n, k, T, L, and q are the ideality factor, Boltzmann constant, absolute temperature, light intensity, and elementary charge, respectively.





**Figure S1.** The calculated binding energy of different structure.





**Figure S2.** Liquid UV-vis of perovskite precursor.


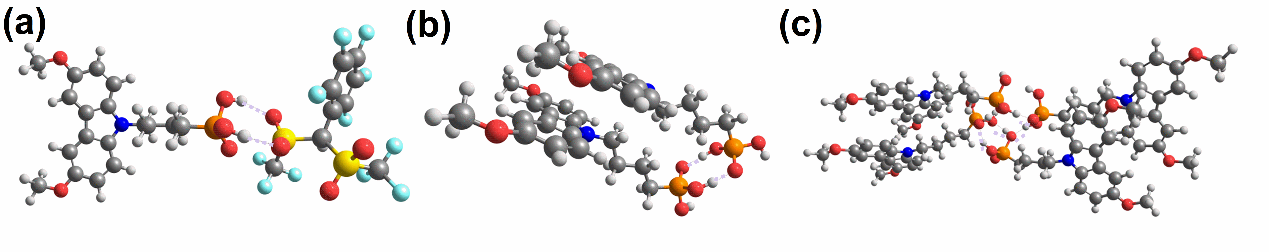


**Figure S3.** The Optimized structures of the (a) TFSP-MeO-4PACz dimer, (b) MeO-4PACz dimer and (c) MeO-4PACz tetramer observed in MD simulations. Critical intermolecular distances are highlighted aside from the structure and the binding energy is given below.


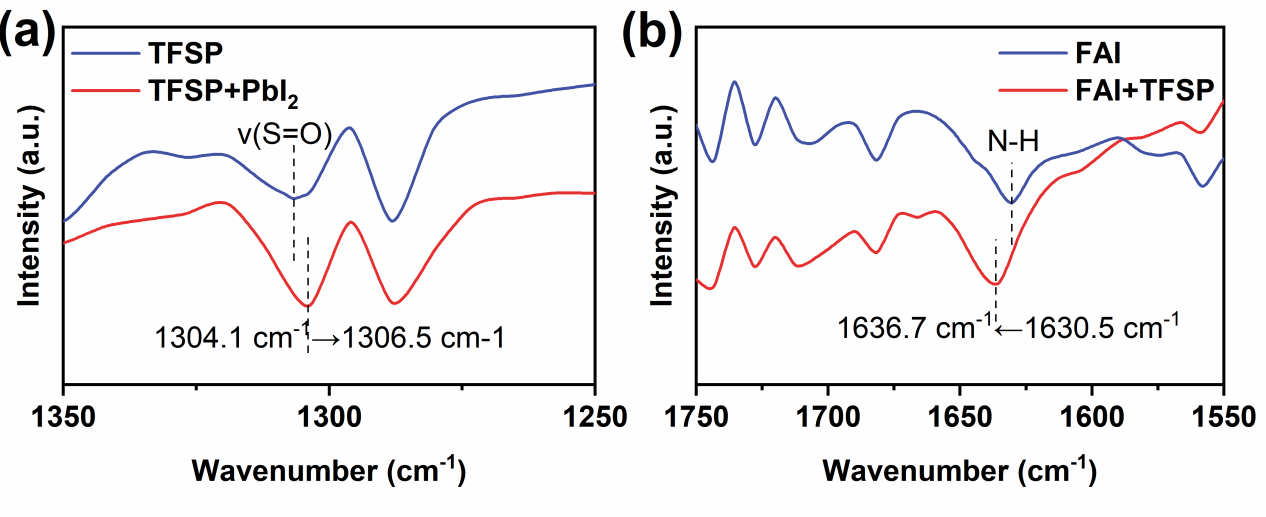


**Figure S4.** FT-IR spectra of (a) the TFSP and TFSP+PbI_2_ flims and (b) the FAI and FAI+TFSP films.


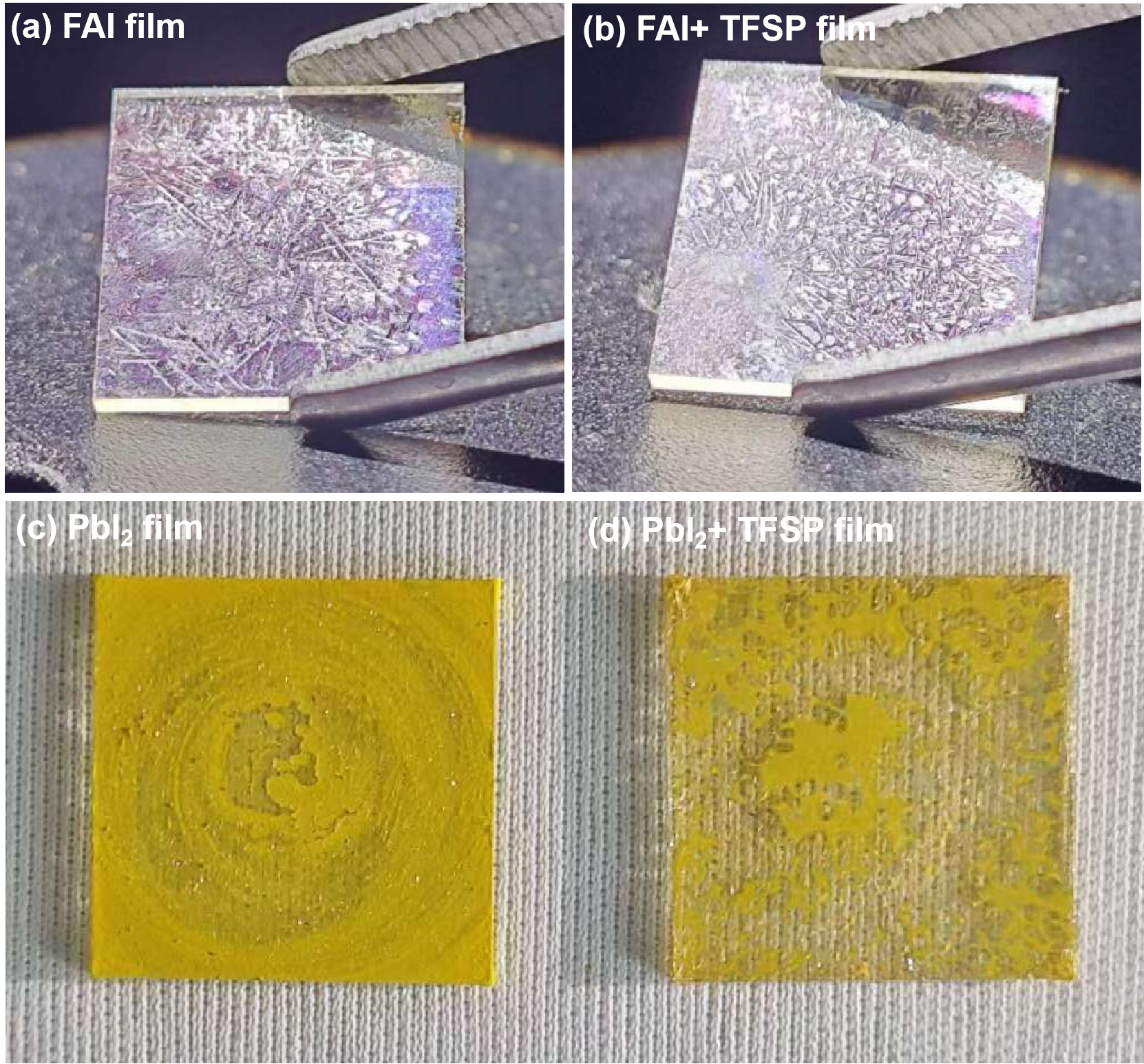


**Figure S5.** FAI films and PbI_2_ films of control and target samples.





**Figure S6.** XPS patterns of the C 1s, F 1s, Pb 4f, I 3d for FAI, PbI_2_ and perovskite (PVK) films.





**Figure S7.** In situ UV-vis of (a) control and (b) target perovskite film in spin-coating and annealing stage.







**Figure S8.** Distribution maps of perovskite grains for the (a) control and (b) target perovskite films.


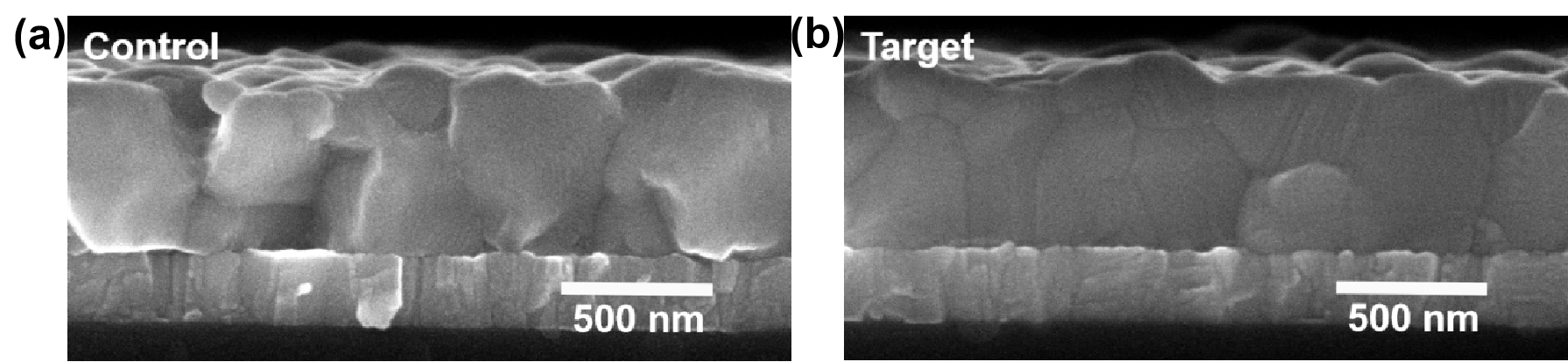


**Figure S9.** Cross section SEM of (a) control and (b) target perovskite films.


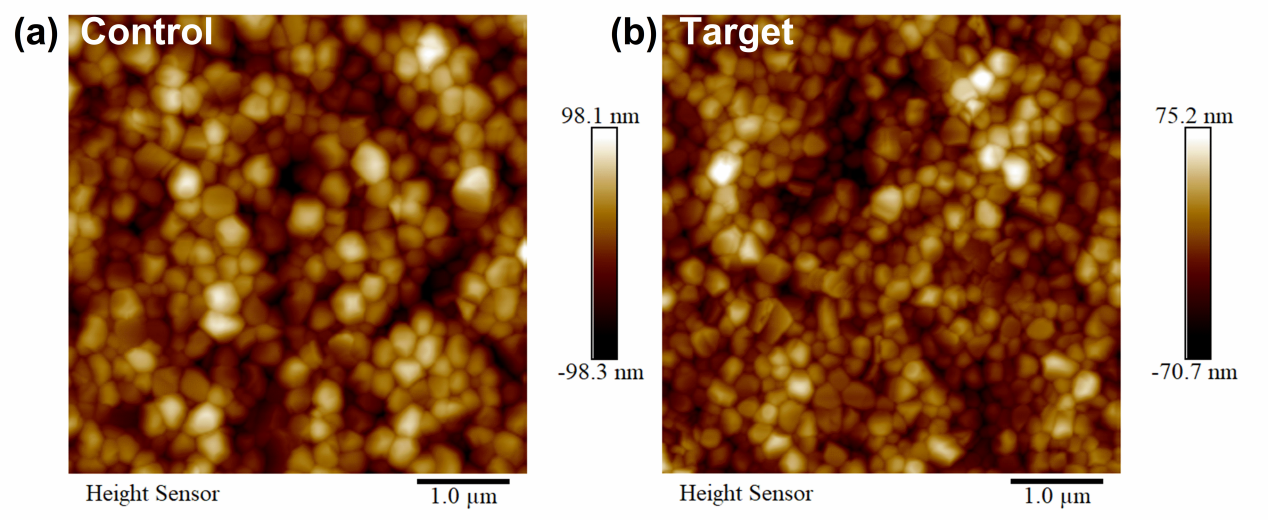


**Figure S10.** AFM images of the (a) control (R_q_: 28.7nm) and (b) target (R_q_: 20.1nm) thin films.


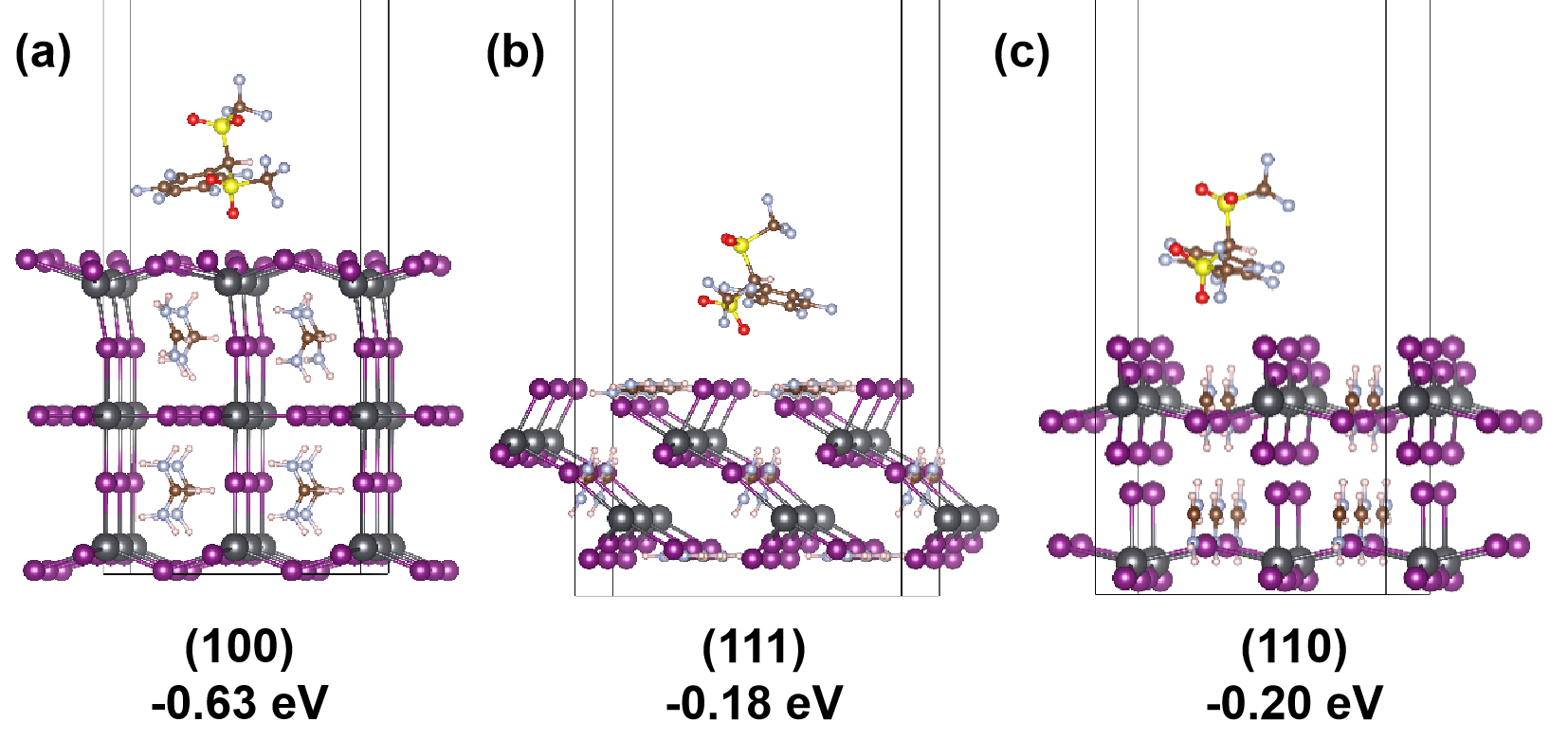


**Figure S11.** Binding energy of (a) (100), (b) (110), (c) (111) of FAPbI_3_.


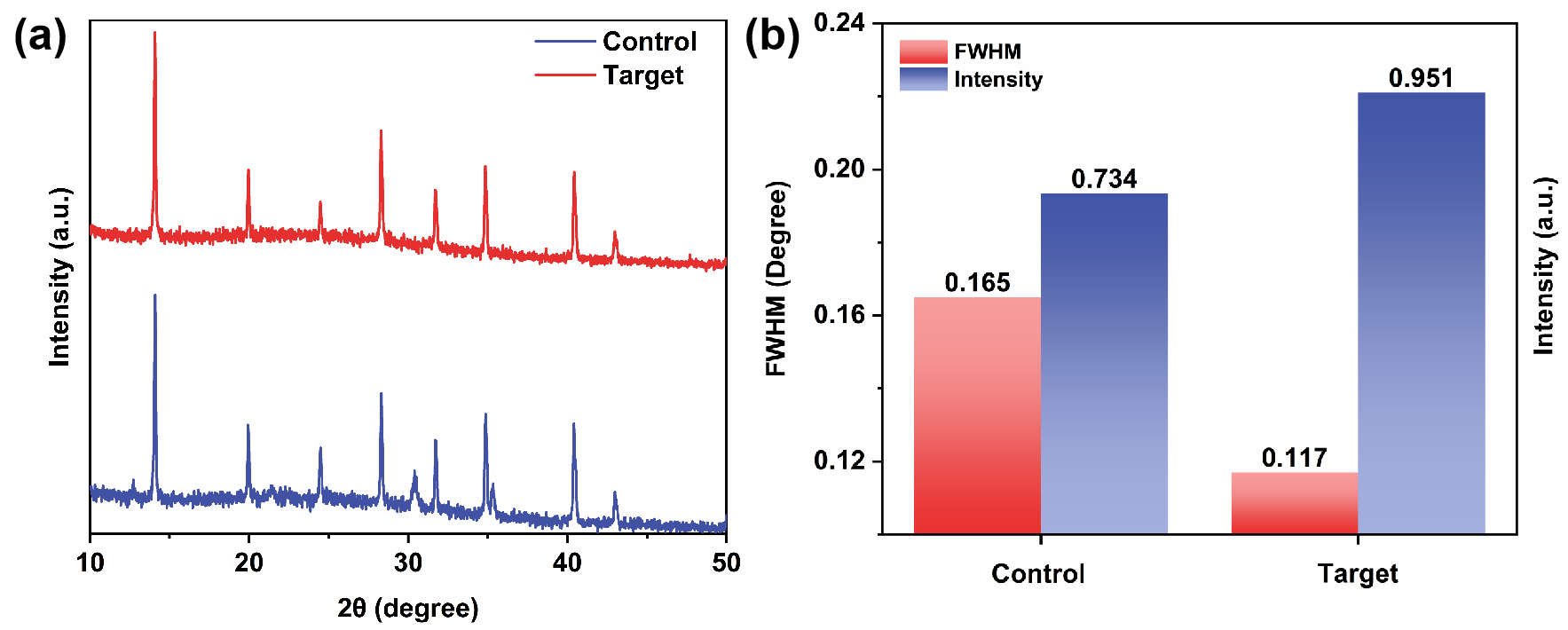


**Figure S12.** (a) XRD pattern and (b) (100) FWHM and intensity of control and target perovskite films.


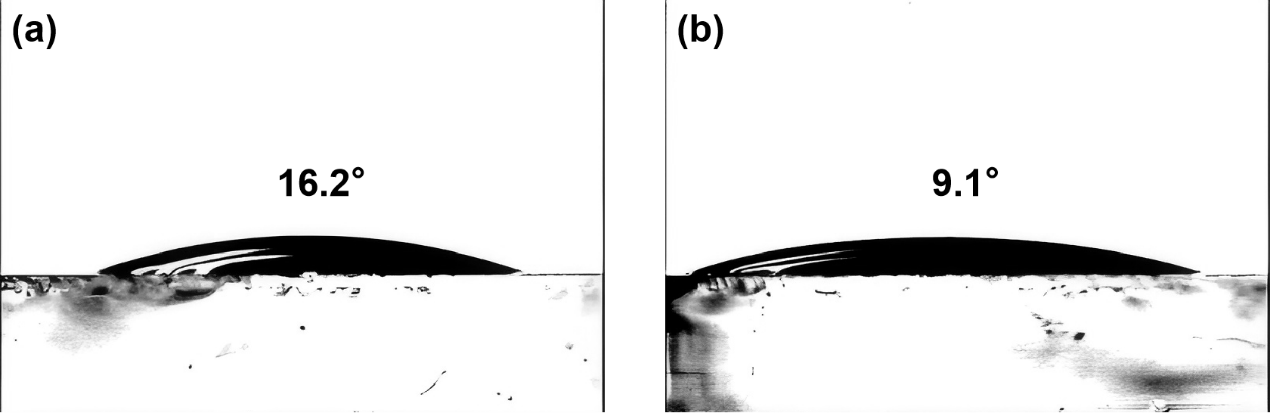


**Figure S13.** XRD Contact angle of (a) control and (b) target perovskite precursor on MeO-4PACz substrates.



**Figure S14.** The conductivity of (a) MeO-4PACz and MeO-4PACz/TFSP films, (b) PCBM and PCBM/TFSP films.





**Figure S15.** The Hall effect of (a) MeO-4PACz and MeO-4PACz/TFSP films, (b) PCBM and PCBM/TFSP films.





**Figure S16.** PL spectra of (a) the ITO/MeO-4PACz/Perovskite structure without and with TFSP treatment, (b) the ITO/Perovskite/PCBM structure without and with TFSP treatment.





**Figure S17**. The fitting lines of 2θ–sin^2^ ψ of GIXRD.





**Figure S18.**The integrated intensity of GIWASX.





**Figure S19**. PL mapings of (a) control and (b) target perovskite films.





**Figure S20.** TA spectra of (a) control and (b) target film.


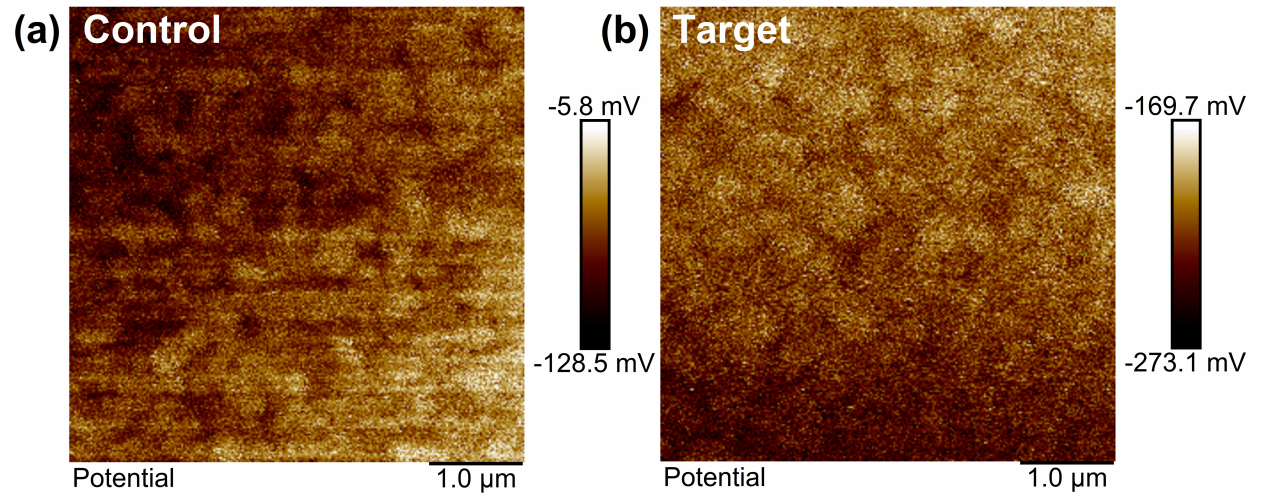


**Figure S21.** KPFM of (a) control and (b) target films.


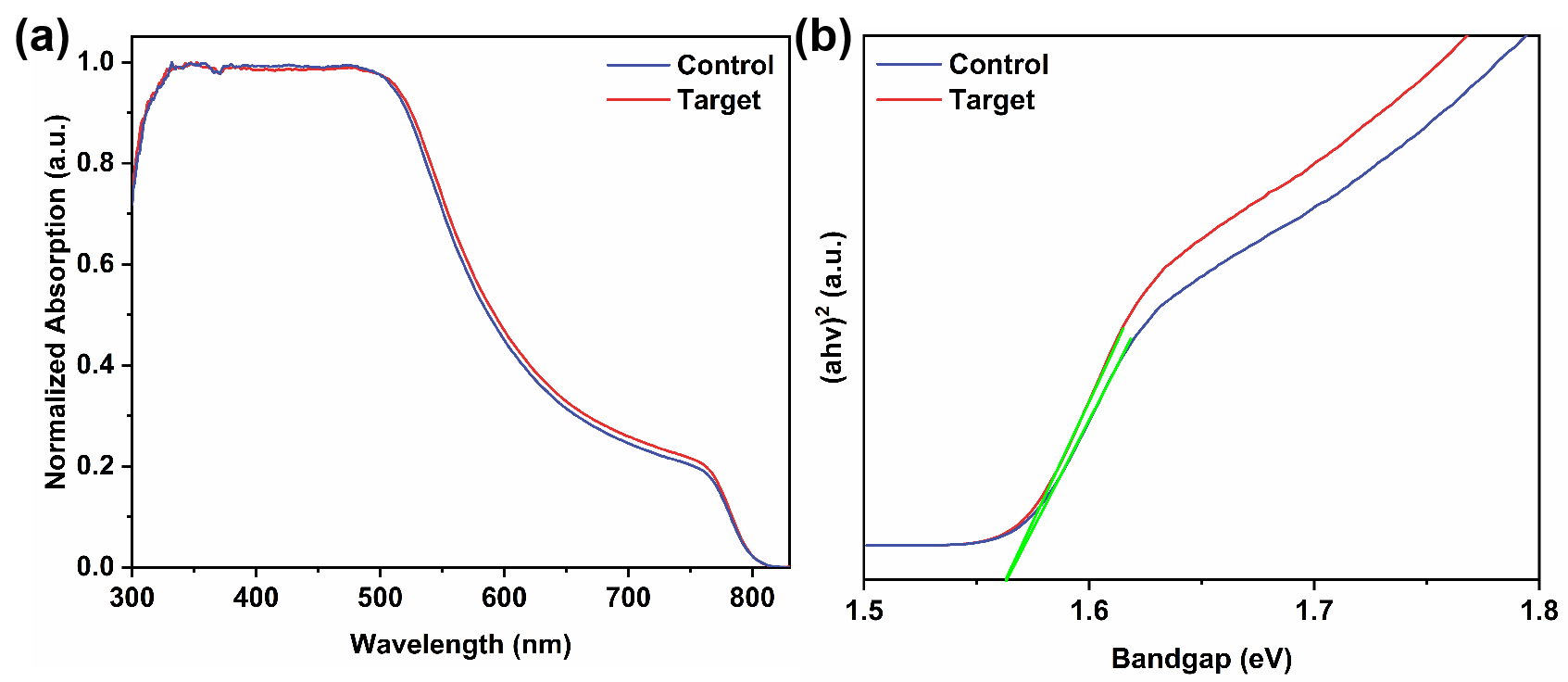


**Figure S22.** (a) UV-vis and (b) Tacu plot of control and target perovskite films.

**
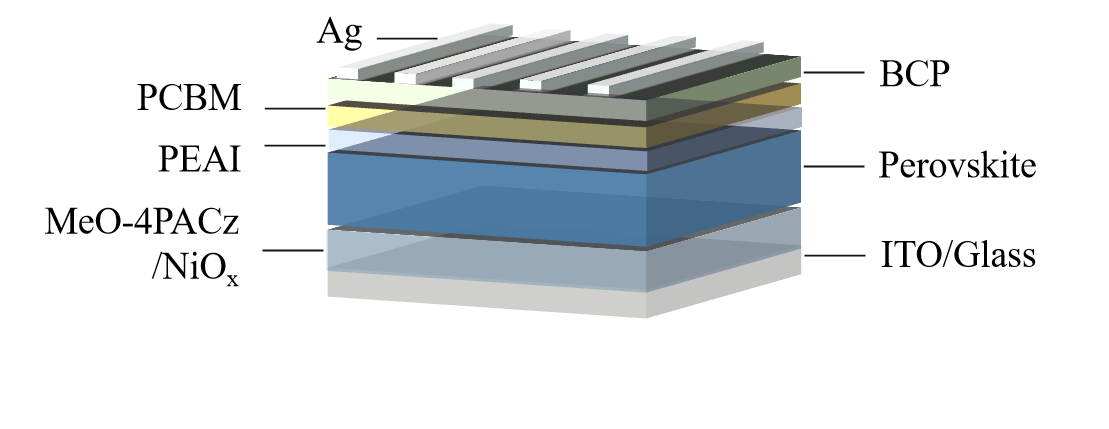
Figure S23.** Device structure diagram.





**Figure S24.** Statistical distribution of the (a) PCE, (b) V_OC_, (c) J_SC_, and (d) FF of the control and target devices.





**Figure S25.** Hysteresis of the (a) control and (b) target devices.





**Figure S26.** Dark condition J-V curves of control and target devices.





**Figure S27.** The M−S plot of the control and target PSCs.


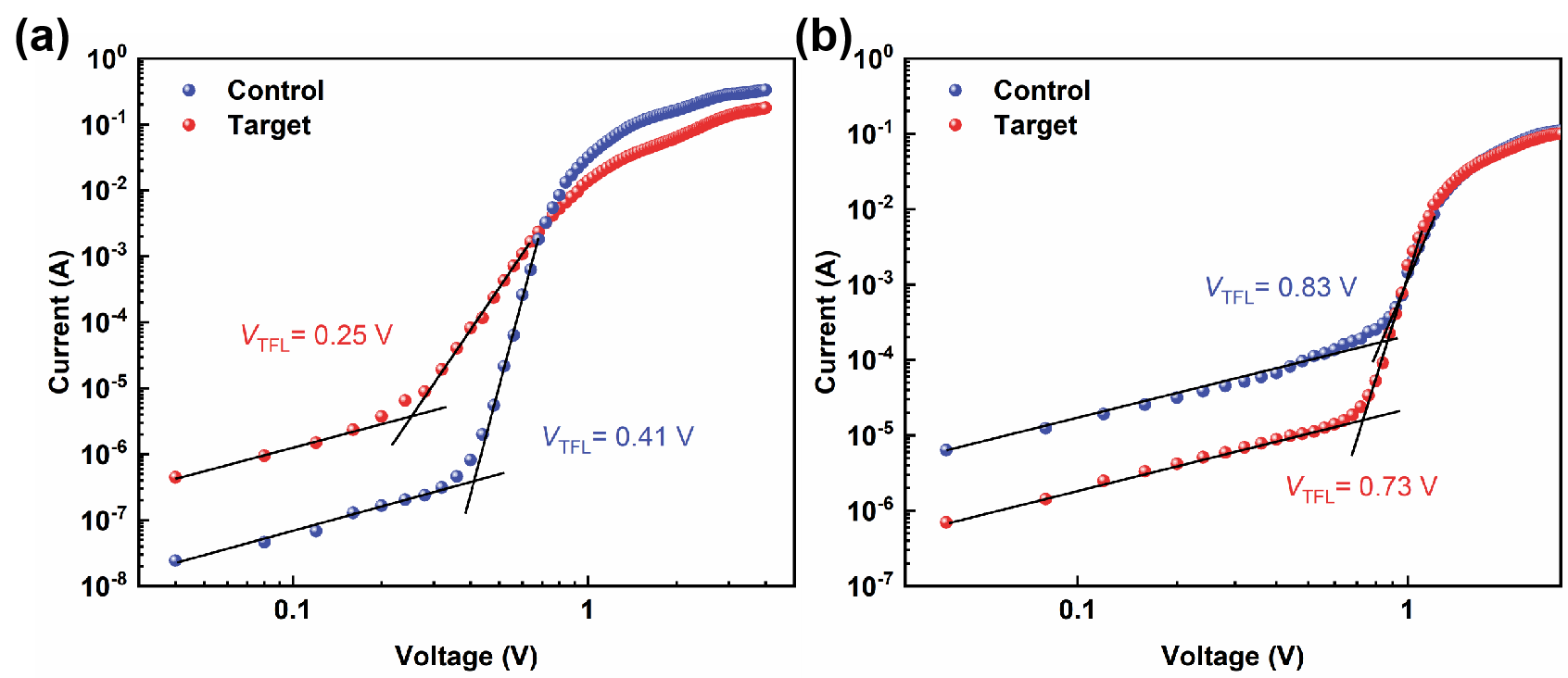


**Figure S28**. SCLC measurement of the (a) electron-only and (b) hole-only devices.





**Figure S29**. Influence of the light intensity on J_SC_ of PSCs.


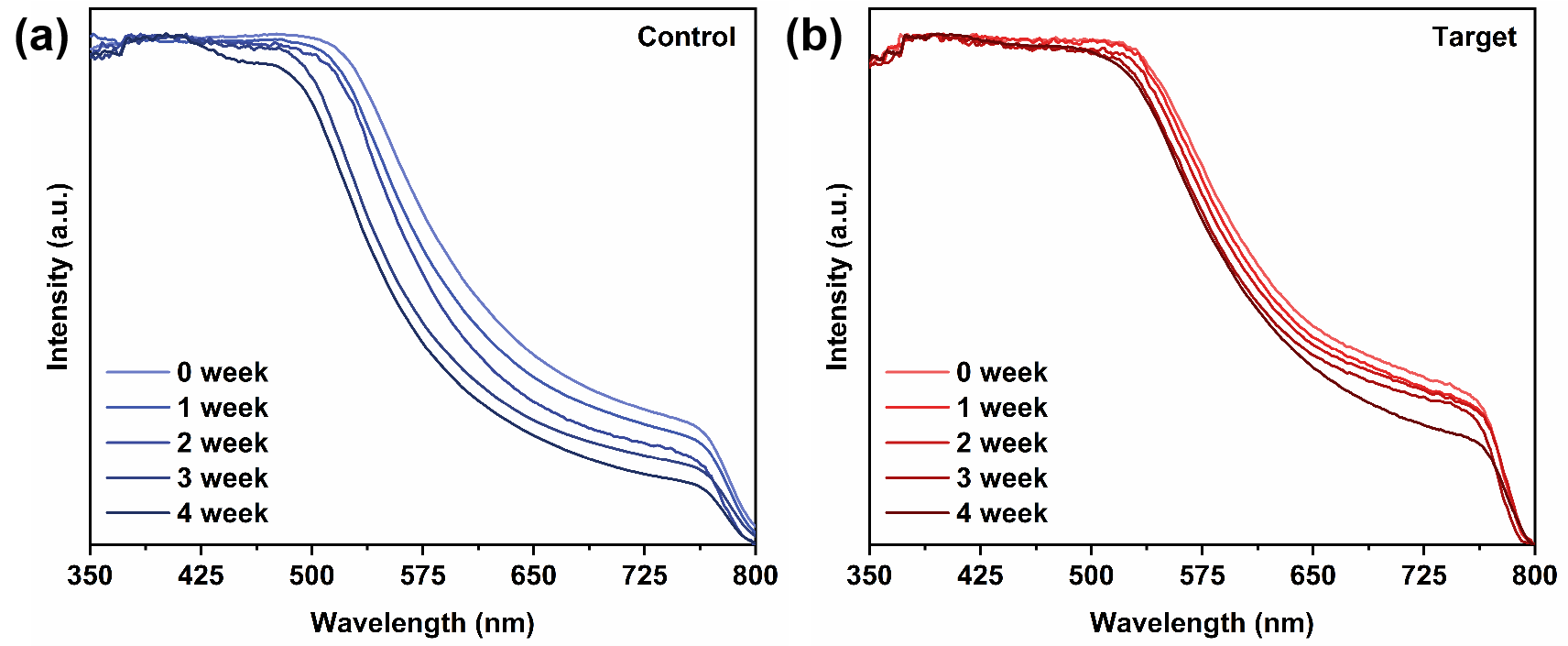


**Figure S30**. The UV-vis spectrum curves of ageing (a) control and (b) target perovskite films measured at 25°C under continuous light illumination at 100 mW·cm^-2^ in ambient conditions for four weeks.


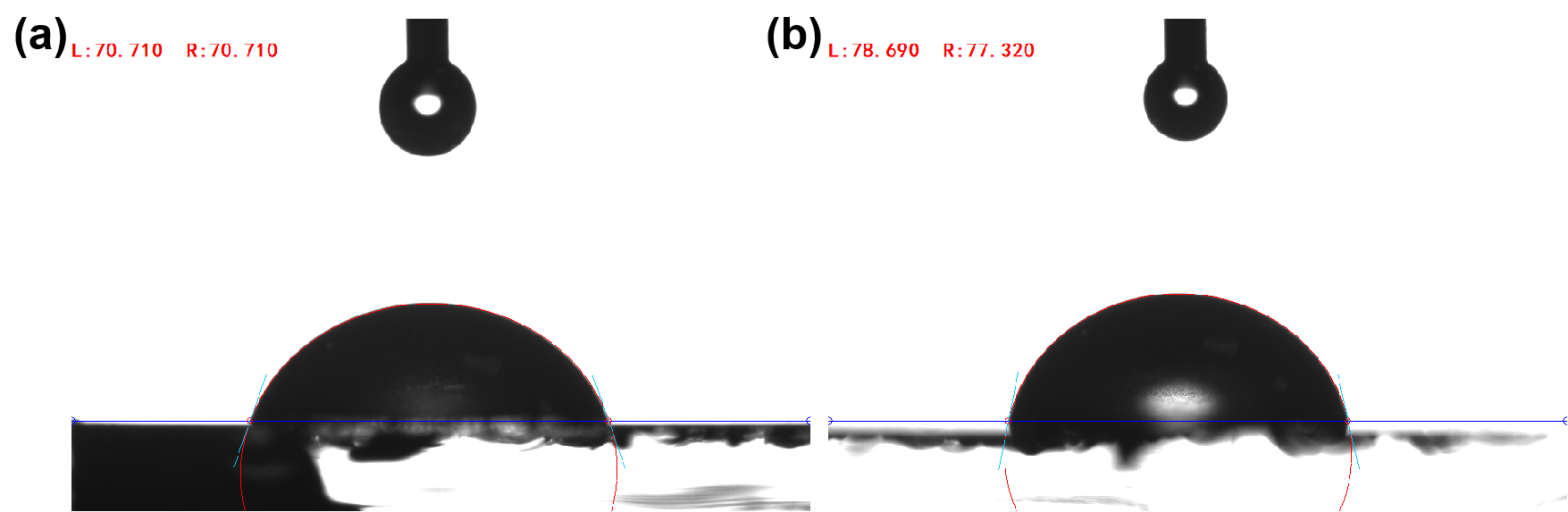


**Figure S31.** The contact angles of water on (a) control and (b) target perovskite films.

**Table S1.** Bandgap and energy level position versus vacuum energy level for the MeO‒4PACz, the perovskite active layer based, and PCBM.

|  | WF (eV) | VBM (eV) | CBM (eV) |
| --- | --- | --- | --- |
| MeO‒4PACz | ‒5.11 | ‒5.29 | ‒2.20 |
| Perovskite | ‒4.59 | ‒5.59 | ‒4.04 |
| Perovskite + TFSP | ‒4.68 | ‒5.77 | ‒4.15 |
| PCBM | ‒4.77 | ‒6.00 | ‒4.22 |

**Table S2.** Parameters of the *J-V* of control, and target PSCs in Figure S23

| Devices | Scan | *V*_oc_ (V) | *J*_sc_ (mA/cm^2^) | FF (%) | PCE (%) |
| --- | --- | --- | --- | --- | --- |
| Control | Reverse | 1.113±0.003 | 25.28±0.45 | 82.07±1.00 | 23.15±0.38 |
|  | Forward | 1.112±0.003 | 25.21±0.51 | 81.07±1.21 | 22.71±0.44 |
| Target | Reverse | 1.114±0.003 | 26.31±0.22 | 85.34±0.89 | 25.17±0.29 |
|  | Forward | 1.113±0.003 | 26.10±0.33 | 85.56±0.83 | 25.08±0.32 |

**Table S3.** Parameters of the *J-V* of control, and target PSCs in Figure S24

| Devices | Scan | *V*_oc_ (V) | *J*_sc_ (mA/cm^2^) | FF (%) | PCE (%) |
| --- | --- | --- | --- | --- | --- |
| Control | Reverse | 1.112 | 25.59 | 82.11 | 23.37 |
|  | Forward | 1.111 | 25.21 | 81.07 | 22.71 |
| Target | Reverse | 1.114 | 26.37 | 85.85 | 25.22 |
|  | Forward | 1.113 | 26.10 | 85.56 | 24.86 |

**Table S4.** The calculated carrier mobility base on SCLC curves of hole only and electron only devices.

|  | J/V^2^ (mA/cm^2^·V^2^) | μ (cm^2^/V·S) |
| --- | --- | --- |
| HTL/Perovskite | 59.73 | 116 |
| HTL/Perovskite+TFSP | 72.10 | 141 |
| Perovskite/ETL | 70.42 | 138 |
| Perovskite+TFSP/ETL | 99.67 | 195.5 |
